# The Pharmaceutical Industry in 2017. An Analysis of FDA Drug Approvals from the Perspective of Molecules

**DOI:** 10.3390/molecules23030533

**Published:** 2018-02-27

**Authors:** Beatriz G. de la Torre, Fernando Albericio

**Affiliations:** 1 KRISP, College of Health Sciences, University of KwaZulu-Natal, Durban 4001, South Africa; 2 School of Chemistry and Physics, University of KwaZulu-Natal, Durban 4001, South Africa; 3 CIBER-BBN, Networking Centre on Bioengineering, Biomaterials and Nanomedicine, Department of Organic Chemistry, University of Barcelona, 08028 Barcelona, Spain

**Keywords:** drug discovery, API, peptide, biologics, small molecules, chemical entities

## Abstract

This is an analysis from a chemical point of view of the 46 drugs (34 New Chemical Entities and 12 Biologics) approved by the FDA during 2017. The drugs included in the 2017 “harvest” have been classified on the basis of their chemical structure: biologics (antibodies and proteins); peptides; amino acids and natural products; drug combinations; and small molecules.

Taking the last 20 years as a reference, 2017 witnessed a record in terms of new entities approved by the FDA: 34 New Chemical Entities (NCEs) (see Figure 1) and 12 biologics [1]. This number is close to the peak reached in 1996, when 59 entities (53 NCEs and 6 biologics) were approved, and much greater than the numbers achieved in recent years [1]. Furthermore, the numbers in 2017 represent a breakthrough when compared with 2016, when only 22 (15 + 7) entities were approved. However, and as occurred in the previous year, analysts interpret the 2017 figures with caution since the launch of a new drug onto the market involves many variables that are difficult to predict [2,3].

The number of biologics approved (12) in 2017 implies the consolidation of this kind of drug. This excellent number is further supported by the six approvals granted by the Center for Biologics Evaluation and Research (CBER), including the first gene therapy treatment [2,4].

Of the 12 biologics approved in 2017, nine are antibodies, one is an antibody-drug conjugate (ADC), and two are enzymes (Table 1).

It is important to draw attention to the approval of the ADC, Besponsa^TM^, for the treatment of acute lymphoblastic leukemia. In 2016, no such compound was approved. Besponsa can be considered a second generation of Mylotarg^TM^ (gemtuzumab ozogamicin), which, in 2000, was the first ADC approved by the FDA for the treatment of acute myeloid leukemia [5]. In 2010, Mylotarg^TM^ was withdrawn from the market because, when combined with chemotherapy, it did not enhance survival and it showed greater toxicity than the chemotherapy alone. However, Mylotarg^TM^ was approved again by the FDA in September 2017 for the treatment of acute myeloid leukemia whose tumors express the CD33 antigen. Analysts forecast that more ADCs will be approved by the FDA in the coming years [6].

With regard to TIDES (oligonucleo- and pep-TIDES), while 2016 was an excellent year for oligonucleotides (three were approved) [7], 2017 was exceptional for peptides (six approved in comparison with one in 2016) (Figure 2). From a structural point of view, the six peptides cover almost the whole spectrum of complexity. Angiotensin II (Giapreza^TM^) is a simple linear octapeptide for the control of blood pressure in adults with sepsis or other critical conditions. Etelcalcetide (Parsabiv^TM^), for the treatment of secondary hyperparathyroidism, is an intriguing peptide formed by a chain of seven d-amino acids with an acetylated d-Cys at the N-terminal, which forms a disulfide bridge with a l-Cys, which is the only L-amino acid in the peptide. Plecanatide (Trulance^TM^), formed by 16 amino acids and with two disulfide bridges, is indicated for the treatment of chronic idiopathic constipation. Plecanatide has a similar mode of action as Linaclotide (Constella^TM^-Linzess^TM^) (14 amino acids and 3 disulfide bridges), already in the market [8]. Abaloparatide (Tymlos^TM^) is a linear peptide comprising 34 amino acids and contains an aminoisobutyric (Aib) residue. It is a parathyroid hormone-related protein analog for the treatment of osteoporosis. Semaglutide (Ozempic^TM^) is a modified human glucagon-like peptide-1 (GLP-1) analog for the treatment of type 2 diabetes mellitus and it can be administered once a week. Semaglutide is structurally similar to liraglutide, which was approved by the FDA in 2010, where the last residue before the N-terminal Ala is replaced by Aib and the acyl moiety in the side-chain of the Lys contains two mini-PEG amino acids, one Glu residue linked to the chain through the ω-carboxylic group, and a C_18_ diacid.

Particularly interesting is macimorelin (Macrilen^TM^), which has come about from the efforts of Fehrent and Martinez’ group at the University of Montpellier [9]. It is a pseudotripeptide formed by one Aib and two D-Trp, where the C-terminus residue is a formyl gem residue (the amino acid is replaced by a gem diamino moiety, which is formylated at the N-terminus). Macimorelin is used for the treatment of adult growth hormone deficiency. Of note, three of the six peptides approved by the FDA in 2017 contain an Aib residue, which confers enzymatic stability and strengthens the formation of the α-helix, when this is feasible.

Four more drugs have their roots in the field of amino acids (Figure 3). Thus, the core of betrixaban (Bevyxxa^TM^) is the 2-amino-5-methoxybenzoic acid, which is amidated with the 5-chloropyridin-2-amine at the carboxylic group and acylated with the 4-(*N*,*N*-dimethylcarbamimidoyl)benzoic acid at the amino function. Betrixaban is indicated for the treatment of venous thromboembolism. Safinamide (Xadago^TM^), for Parkinson’s disease, is a derivative of the Ala (4-((3-fluorobenzyl)oxy)benzyl)-l-alaninamide. Netarsudil (Rhopressa^TM^), for glaucoma, is a derivative of the β-amino acid (*S*)-3-amino-2-phenylpropanoic acid, which is amidated with 6-amine-isoquinolin. Valbenazine (Ingrezza^TM^), used for tardive dyskinesia, is an ester of L-Val.

The ester part of valbenazine is found in one of the most intriguing drugs approved in 2017, namely Deutetrabenazine (Austedo^TM^) (Figure 4). Of note, this is the first deuterated drug ever approved by the FDA. Deutetrabenazine is the deuterated version (two deuterated methoxy groups) of a generic drug, tetrabenazine. The presence of deuterium is claimed to improve dosing properties and safety. Deutetrabenazine is indicated for chorea associated with Huntington’s disease.

Under the category of drugs inspired by natural products (Figure 5), ertugliflozin (Steglatro^TM^), for diabetes type 2, contains a core of glucose. Deflazacort (Emflaza^TM^), which is indicated for duchenne muscular dystrophy, is a glucocorticoid. Naldemedine (Symproic^TM^), a derivative of noroxymorphone, is used for opioid-induced constipation.

Interestingly, six drugs approved in 2017 contain the 2-amine-pyrimidine moiety (Figure 6). Ribociclib (Kisqali^TM^) for HR-positive/HER2-negative advanced or metastatic breast cancer; brigatinib (Alunbrig^TM^) for ALK-positive non-small-cell lung cancer; abemaciclib (Verzenio^TM^) for breast cancer; copanlisib (Aliqopa^TM^) for follicular lymphoma; telotristat ethyl (Xermelo^TM^) for carcinoid syndrome diarrhea; and letermovir (Prevymis^TM^) for the prevention of infection after bone marrow transplant.

Enasidenib (Idhifa^TM^) is built on a triazine scaffold (Figure 7), which is also present in other drugs, and is indicated for IDH2-positive acute myeloid leukemia.

In 2016, two two-drug combinations for the treatment of hepatitis C, Epclusa^TM^ and Zepatier^TM^ were approved by the FDA. Both contain a hepatitis C virus NSSA inhibitor (velpatasvir and elbasvir, respectively) and sofosbuvir (nucleotide, viral RNA polymerase inhibitor) and grazopevir (macrocycle, NS3/4A protease inhibitor), respectively. In 2017, following the same trend for the treatment of the same disease, two more multi-drug combinations were also approved (Figure 8). Thus, Vosevi^TM^ adds a third drug, voxilaprevir, to the two present in Epclusa^TM^ (velpatasvir and sofosbuvir). Voxilaprevir^TM^ is a NS3/4A protease inhibitor. On the other hand, Mavyret^TM^ combines glecaprevir and pibrentasvir, which are NS3/4A and NS5A protease inhibitors, respectively.

In 2017, one two-drug combination was approved for urinary infections (Figure 9). Vabomere^TM^ combines meropenem, a carbapenem-type antibiotic, and vaborbactam, a cyclic boronic acid pharmacophore that acts as a non-lactam–lactamase inhibitor.

Two antibiotics based on the oxacin moiety (Figure 10), delafloxacin (Baxdela^TM^), which is fluorinated, and ozenoxacin (Xepi^TM^), which is non-fluorinated, were approved, thereby increasing the number of drugs in this family present on the market.

Two drugs are a simple modification of a nitroimidazole moiety (Figure 11). Benznidazole (Benznidazole^TM^), from 2-nitroimidazole, is for Chagas disease. Secnidazole (Solosec^TM^), for bacterial vaginosis, is derived from 5-nitroimidazole. Two more drugs contain a *N*-phenyl-pyrazole structure (Figure 12): niraparib (Zejula^TM^) for fallopian tube, epithelial ovarian, or peritoneal cancer, and edaravone (Radicava^TM^) for amyotrophic lateral sclerosis.

Finally, there are four more small-molecule drugs (Figure 13). The first three for distinct types of cancer: midostaurin (Rydapt^TM^) for FLT3-positive acute myeloid leukemia; neratinib (Nerlynx^TM^) for HER2-positive breast cancer; and acalabrutinib (Calquence^TM^) for Mantle cell lymphoma and, finally, latanoprostene (Vyzulta^TM^) for glaucoma-ocular hypertension.

As mentioned in the introduction, in “Drugs to the Market” it is very difficult to interpret trends and thus make forecasts for the coming years. However, if the approval of seven antibodies and three oligonucleotides were the most notable achievements in recent years, in 2017 the number of antibodies increased to nine and, more importantly, one ADC was approved. These figures serve to support the notion that these molecules will become the “Drugs of the Future” for the treatment of hitherto intractable diseases. It is to be hoped that production methods will evolve to be more cost effective and antibody-based drugs will become affordable for much of the population. This comment also applies to the production of combination drugs to treat hepatitis C, which are currently unaffordable for a large number of patients.

The strength of the privileged structures in the drug discovery process has been clearly highlighted by the approval of six drugs containing 2-amine-pyrimidine moiety, one with a triazine structure, and two with a nitroimidazole.

In 2017, the TIDES section was dominated by peptides. In this regard, six peptides ranging in size from small to large were approved. Five of them are produced using solid-phase technology, thereby showing once again that this simple but efficient approach developed by the Nobel Laureate Bruce Merrifield is a potent tool for drug discovery [10].

Although cancer drugs received the most approvals in 2017, it is important to highlight that several drugs against infectious diseases were also authorized.

The outputs from 2017 indicate that totally synthetic (from the idea) small molecules are losing ground against biologics, biomolecules, and other molecules inspired by natural products. However, all drugs, regardless of the chemical species involved, will be seen to achieve their mission if they are able to relieve suffering. There is hope that the figures for 2018 will exceed those of 2017.

## Figures and Tables

**Figure 1:**
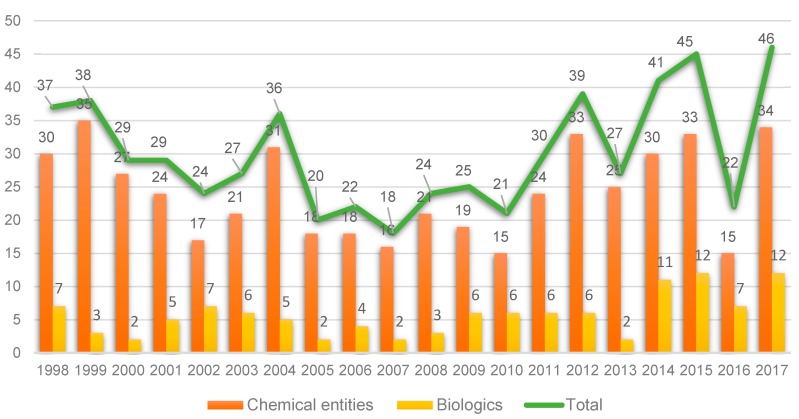
New Chemical Entities and Biologics approved by the FDA in the last two decades [1,2].

**Figure 2:**
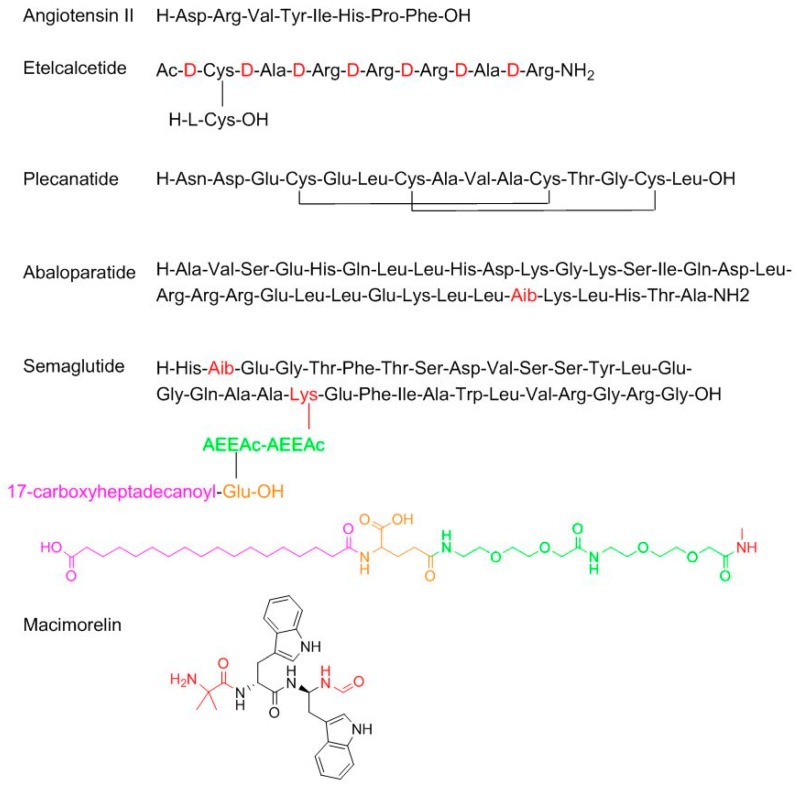
Structure of peptides. In red the non-proteinogenic or modified amino acids.

**Figure 3:**
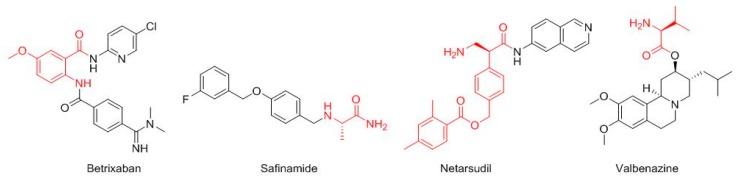
Structure of amino acid-based drugs. In red the amino acid.

**Figure 4:**
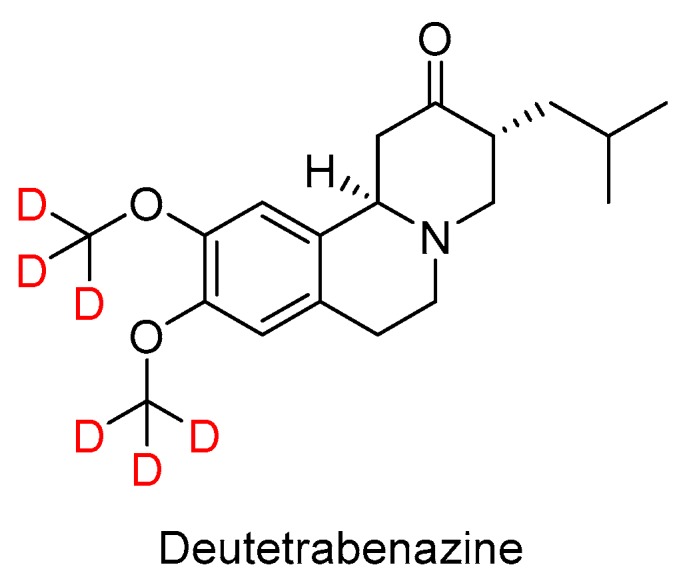
Structure of deutetrabenazine.

**Figure 5:**
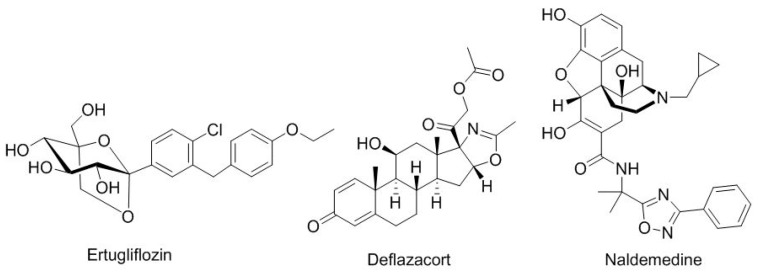
Structure of drugs inspired by natural products.

**Figure 6:**
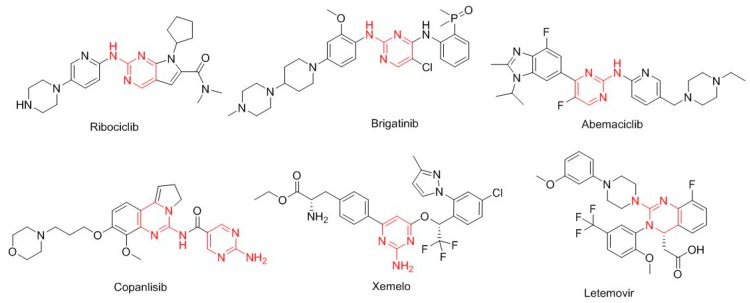
Structure of drugs that contain the 2-amine-pyrimidine (in red) moiety.

**Figure 7:**
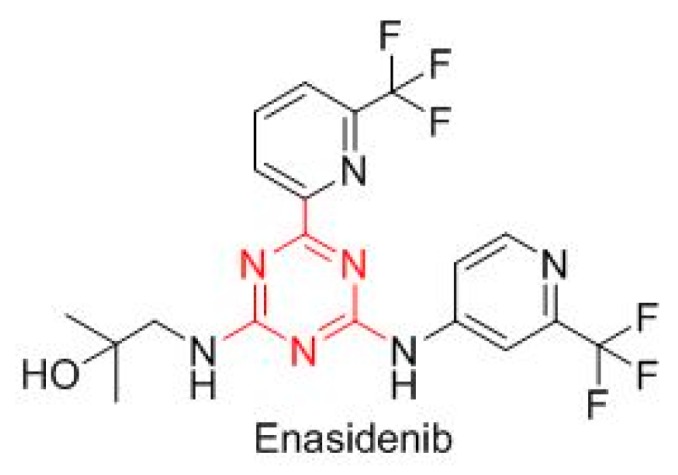
Structure of enasidenib that contains the triazine (in red) scaffold.

**Figure 8:**
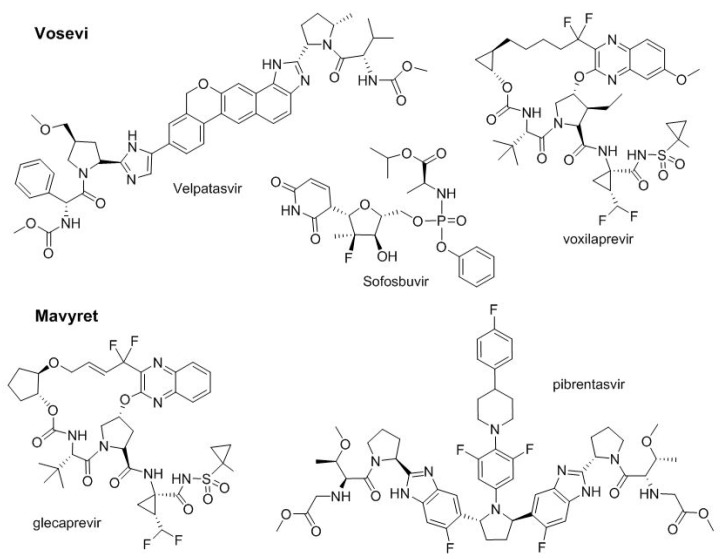
Structure of drug combination for treatment of hepatitis C.

**Figure 9:**
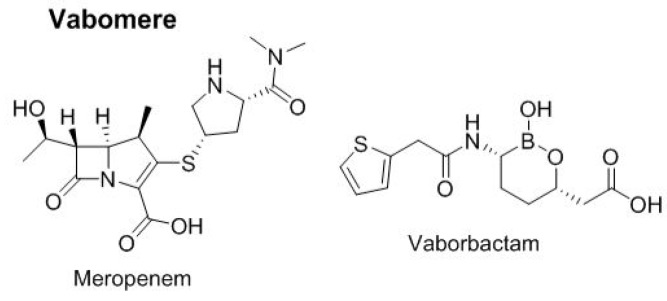
Structure of the drug combination for infections.

**Figure 10:**
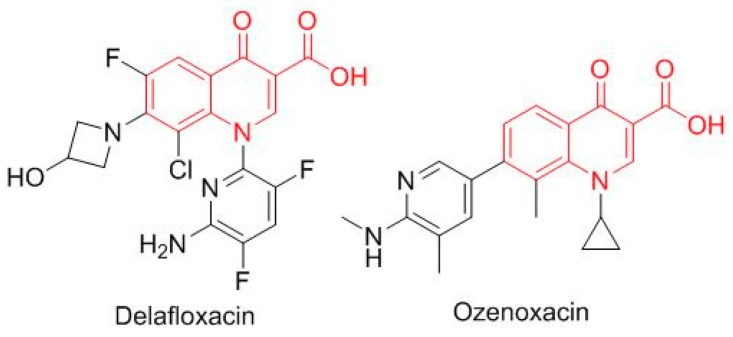
Structure of oxacin (in red) based drugs.

**Figure 11:**
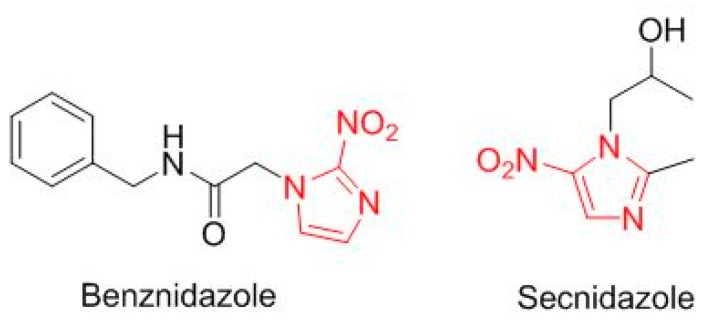
Structure of drugs derived of nitroimidazole (in red).

**Figure 12:**
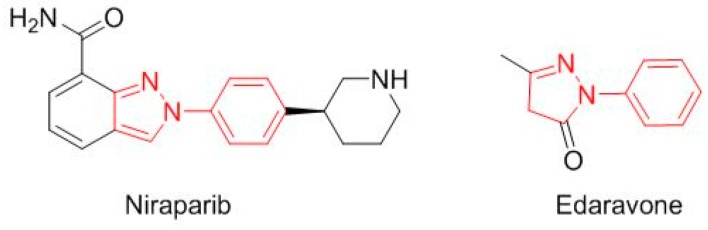
Structure of drugs derived of the *N*-phenyl-pyrazole (in red) structure.

**Figure 13:**
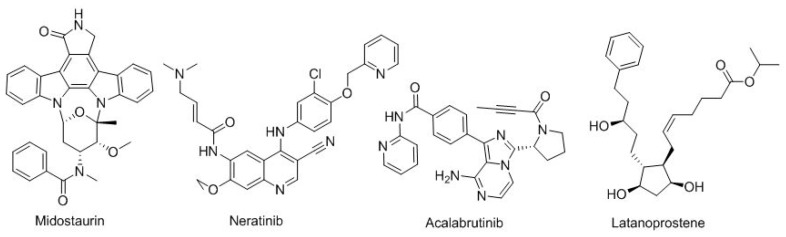
Structure of midostaurin, neratinib, acalabrutinib, and latanoprostene.

**Table 1:** Biologics approved by the FDA in 2017 [1].

Active Ingredient	Trade Name ^a^	Disease
Avelumab	Bavencio^TM b^	Merkel cell carcinoma
Dupilumab	Dupixent^TM b^	Asthma
Benralizumab	Fasenra^TM b^	Asthma
Emicizumab	Hemlibra^TM b^	Hemophilia A
Durvalumab	Imfinzi^TM b^	Urothelial carcinoma
Sarilumab	Kevzara^TM b^	Rheumatoid arthritis
Ocrelizumab	Ocrevus^TM b^	Multiple sclerosis
Brodaluma	Siliq^TM b^	Psoriasis
Guselkumab	Tremfya^TM b^	Psoriasis
Inotuzumab ozogamicin	Besponsa^TM c^	Acute lymphoblastic leukemia
Cerliponase alfa	Dupixent^TM d^	Batten disease
Vestronidase alfa	Fasenra^TM d^	Sly syndrome

^a^ USA; ^b^ antibody; ^c^ ADC; ^d^ enzyme.
